# Supplementary figures and images for: Comparative Analysis Reveals Different Evolutionary Fates and Biological Functions in Wheat Duplicated Genes (Triticum aestivum L.)
Source: Plants (Basel). 2023 Aug 22;12(17):3021. doi: 10.3390/plants12173021 (PMC10489728; doi:10.3390/plants12173021)

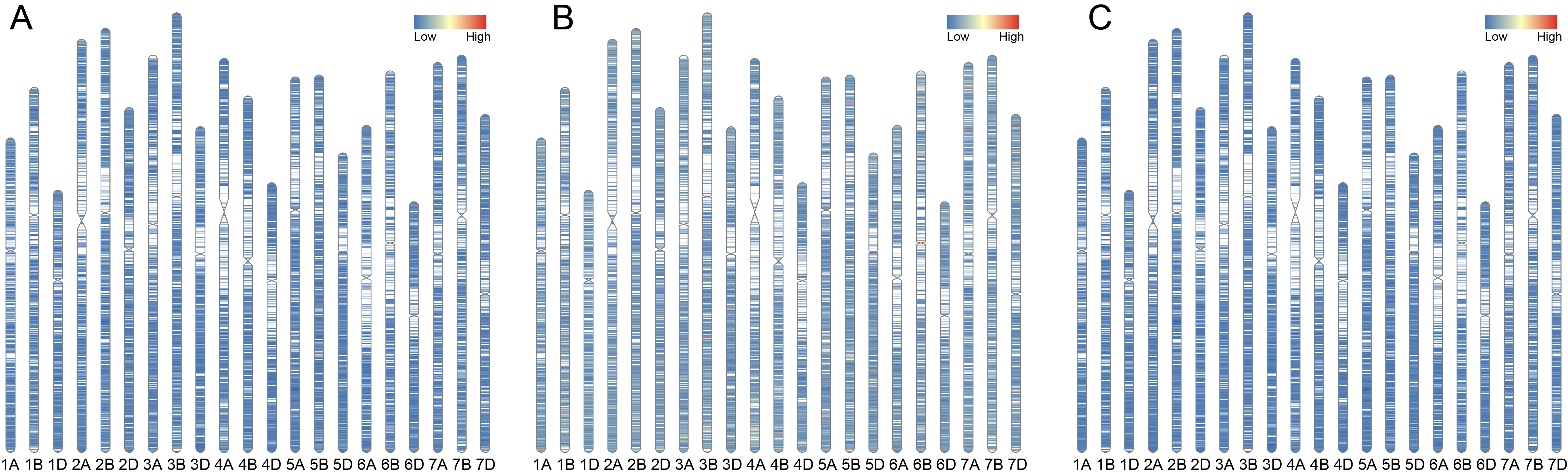

Supplement: Supplementary file 1 [file plants-12-03021-s001.zip › Figure S1.jpg]

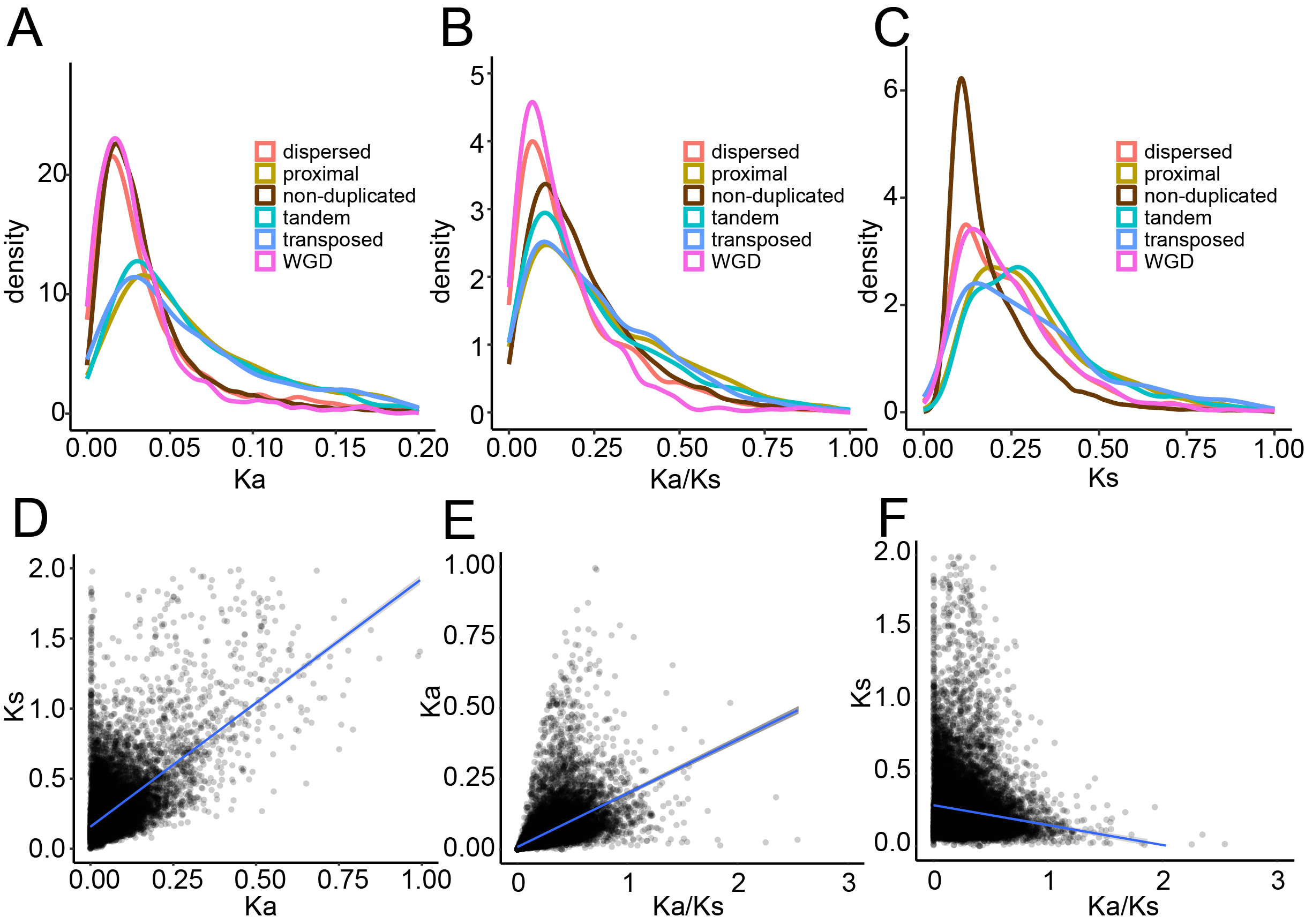

Supplement: Supplementary file 1 [file plants-12-03021-s001.zip › Figure S2.jpg]

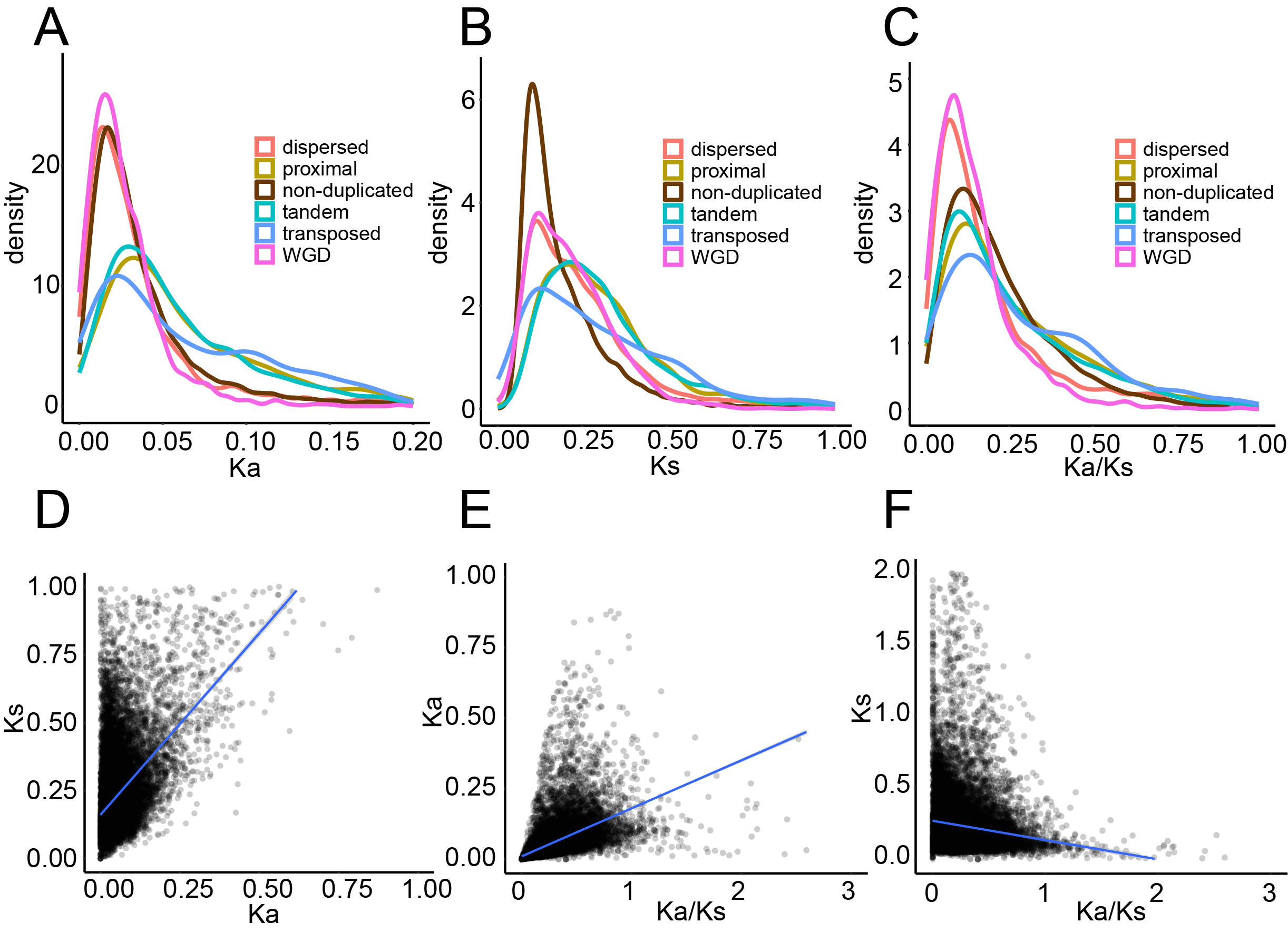

Supplement: Supplementary file 1 [file plants-12-03021-s001.zip › Figure S3.jpg]

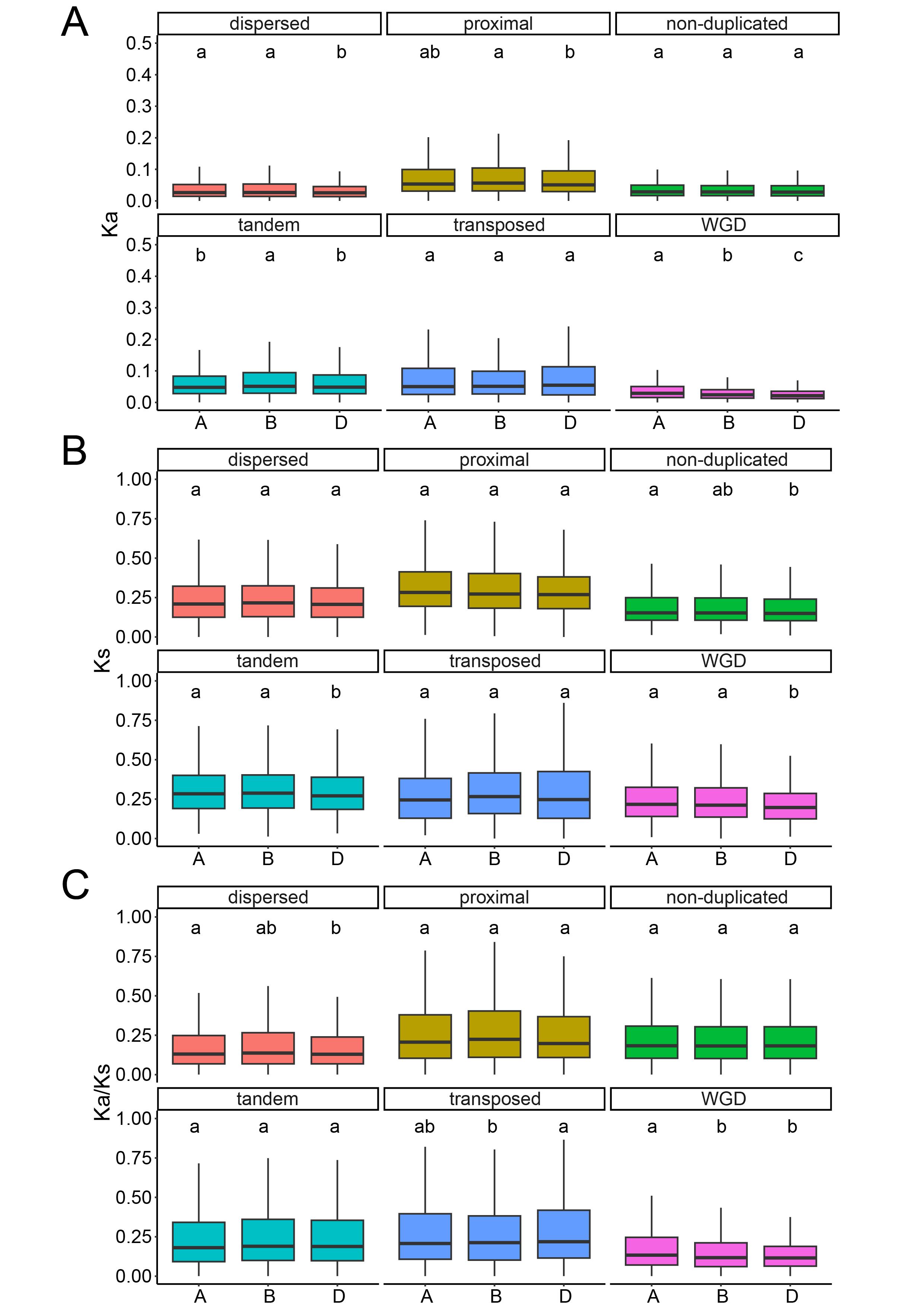

Supplement: Supplementary file 1 [file plants-12-03021-s001.zip › Figure S4.jpg]

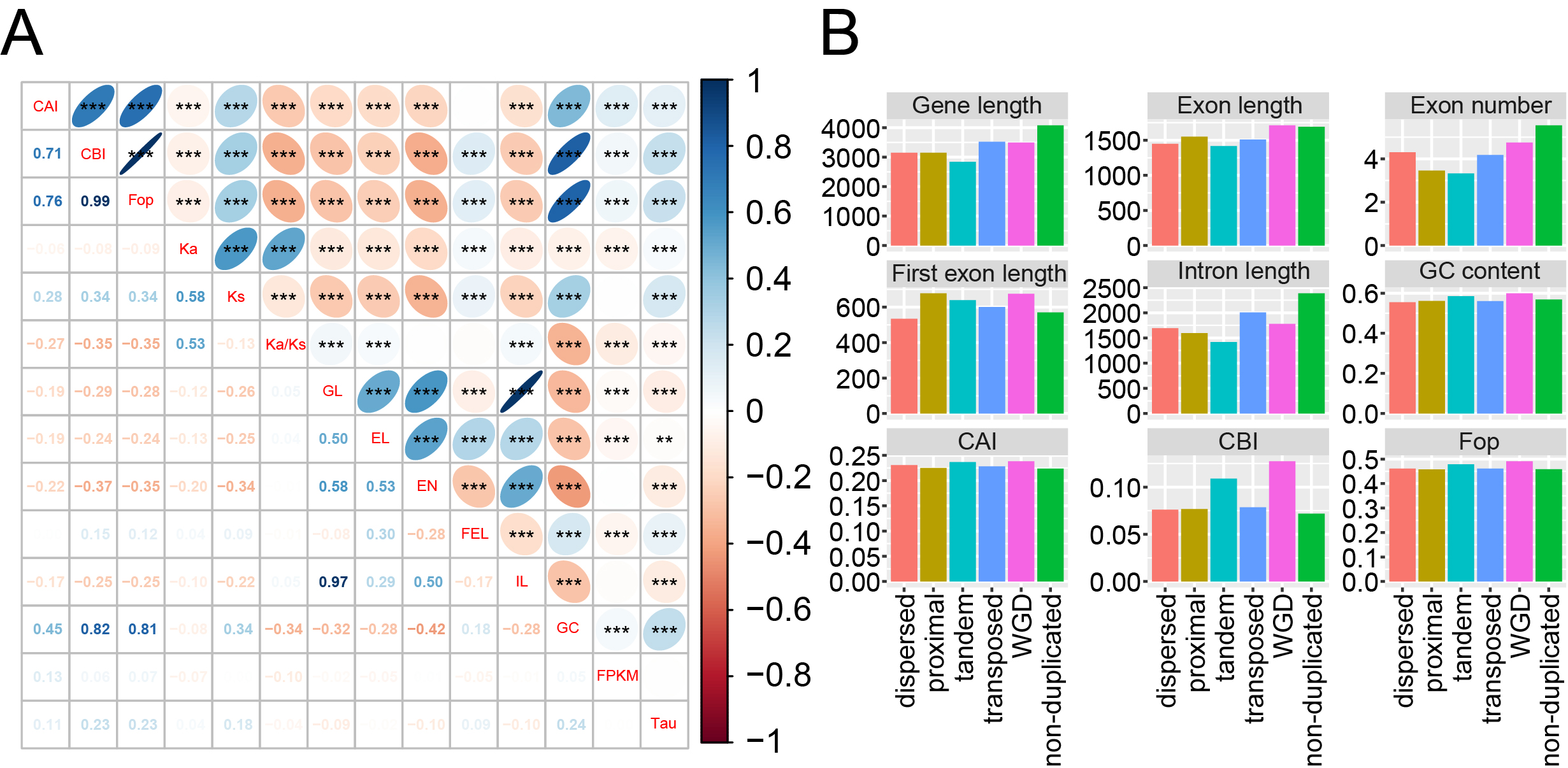

Supplement: Supplementary file 1 [file plants-12-03021-s001.zip › Figure S5.jpg]

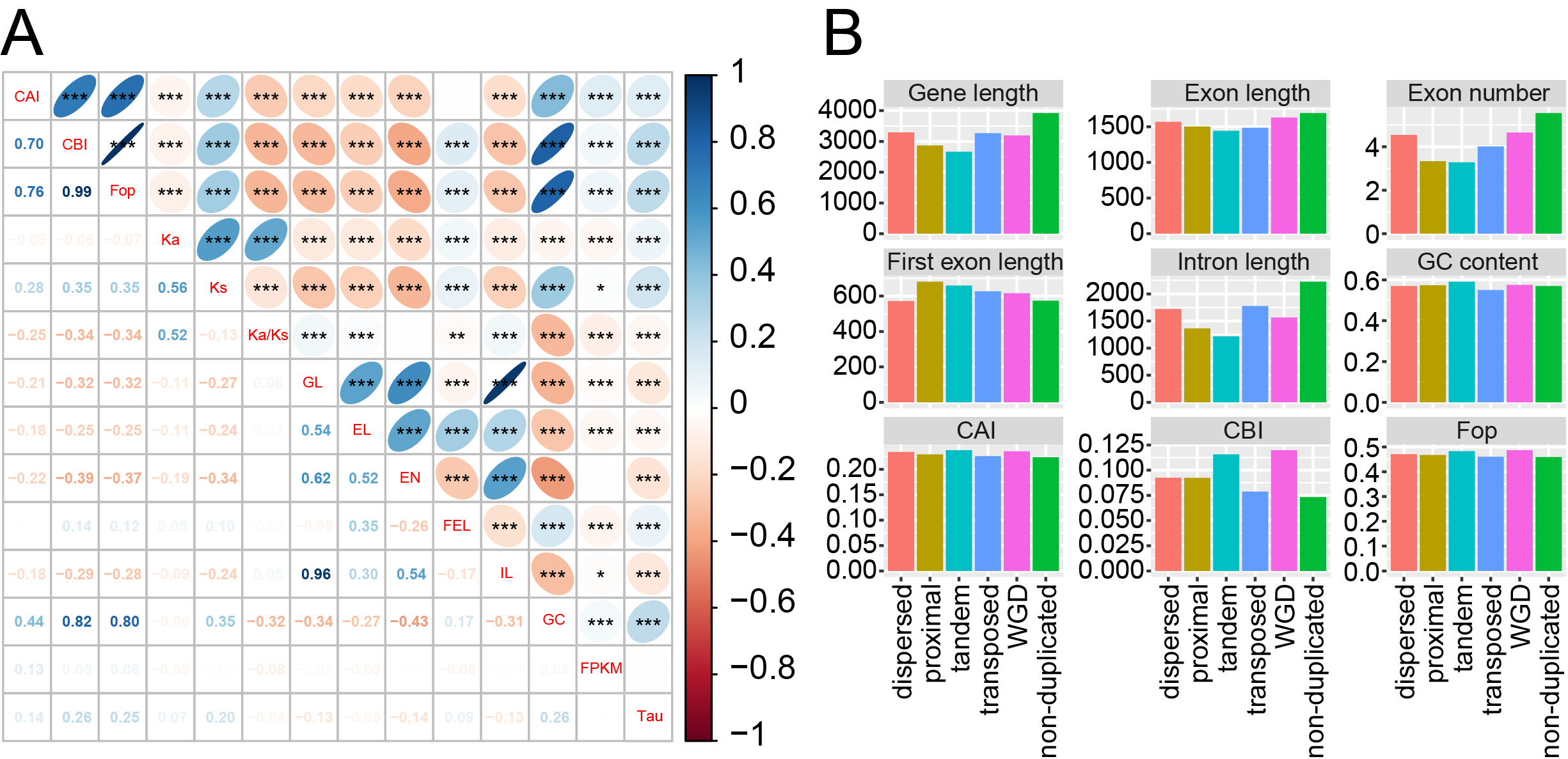

Supplement: Supplementary file 1 [file plants-12-03021-s001.zip › Figure S6.jpg]

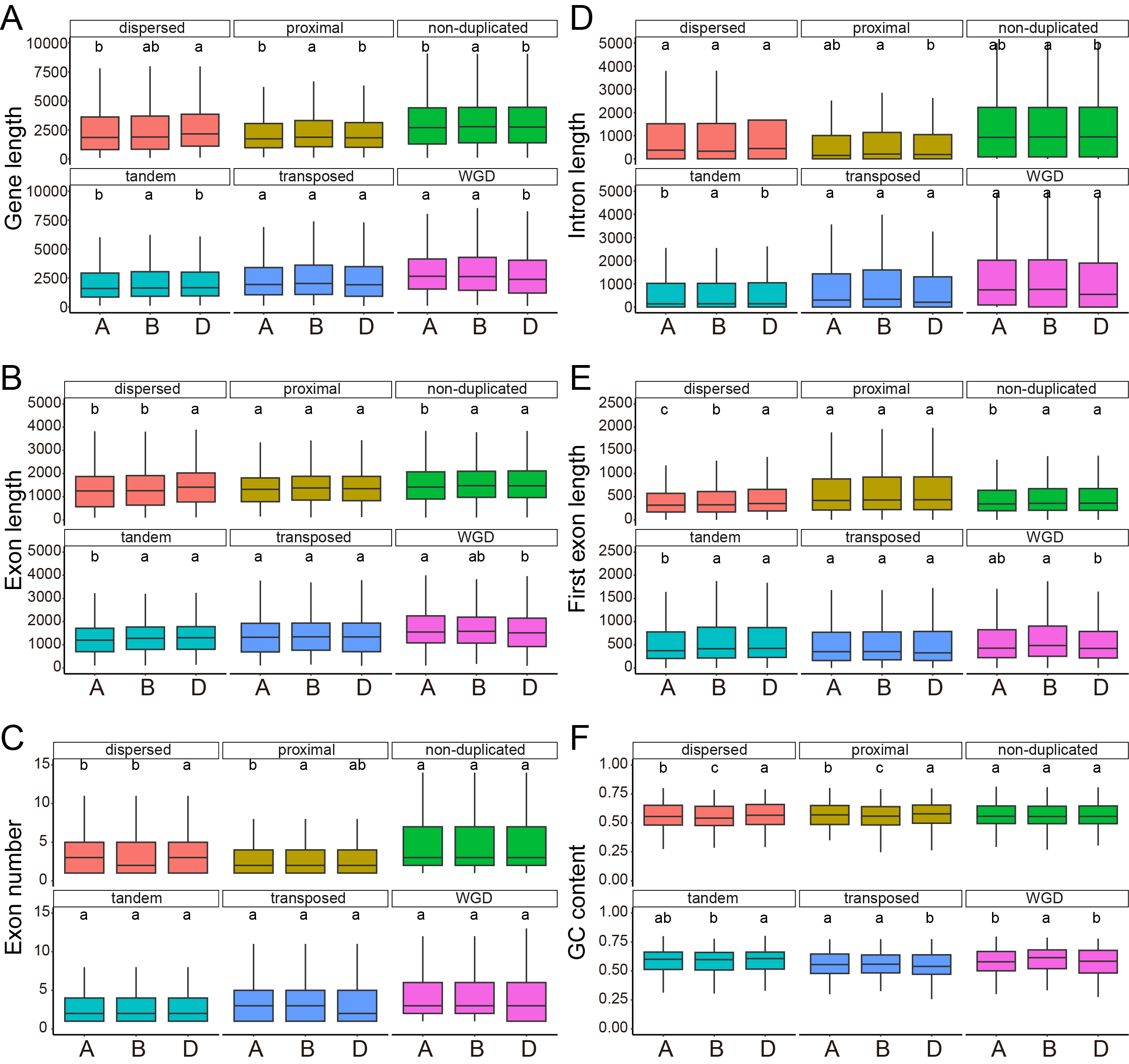

Supplement: Supplementary file 1 [file plants-12-03021-s001.zip › Figure S7.jpg]

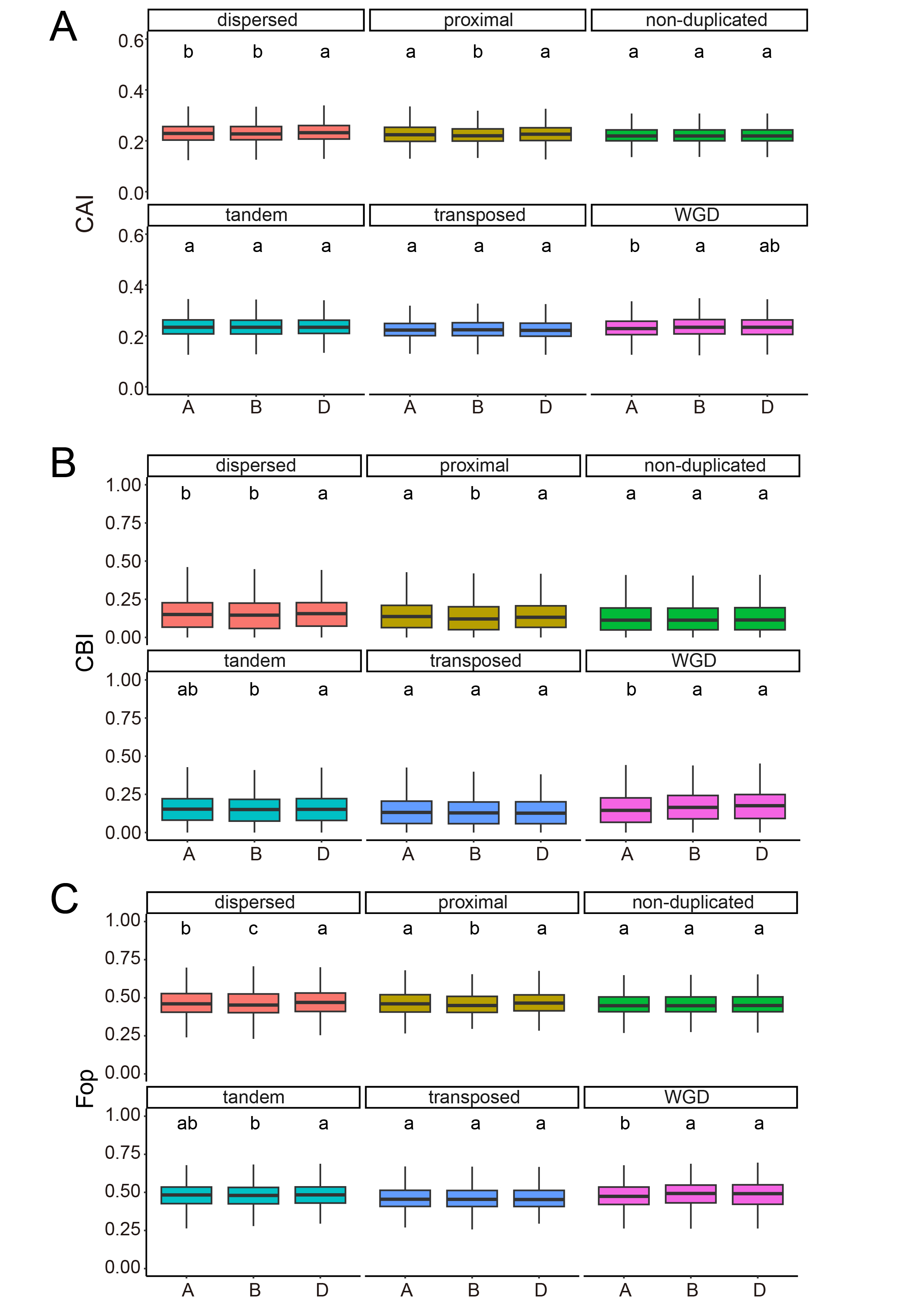

Supplement: Supplementary file 1 [file plants-12-03021-s001.zip › Figure S8.jpg]

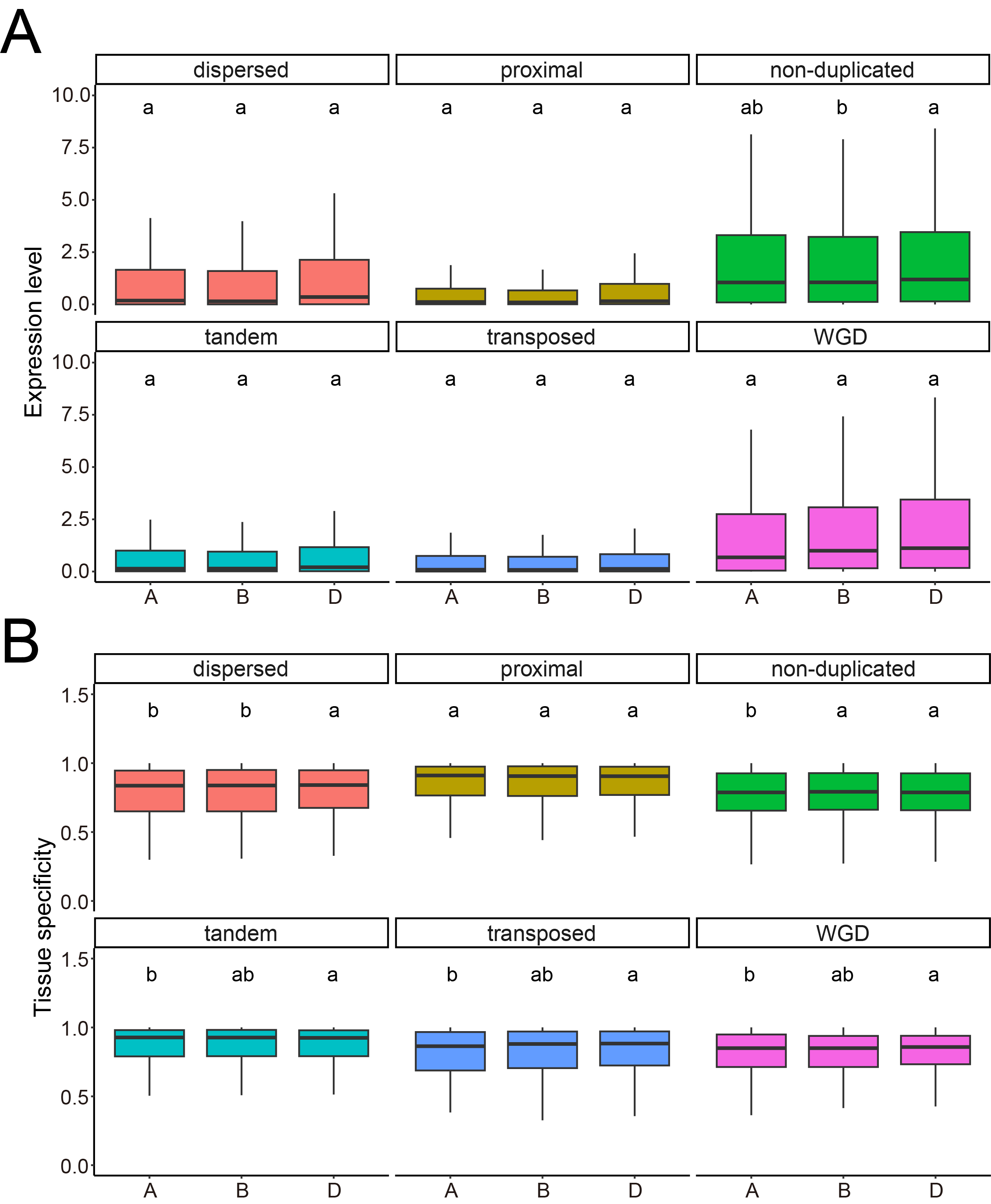

Supplement: Supplementary file 1 [file plants-12-03021-s001.zip › Figure S9.jpg]
